# Remarks on the Use of Clasps for the Retention of Artificial Teeth

**Published:** 1849-07

**Authors:** P. H. Austen

**Affiliations:** Baltimore.


### ARTICLE VI.

*Remarks on the Use of Clasps for the Retention of Artificial Teeth.* By P. H. AUSTEN, M. D., Baltimore.

MESSRS EDITORS.

We should be glad to see some remarks, from the pen of any of the experienced members of the profession, upon the following question: How far may the principle of Cleveland's air cavity be substituted for clasps in the retention of partial pieces of plate work? The question is one of eminent practical importance, and as such, its discussion deserves a place in a journal devoted to the improvement of dental science.

This subject has been suggested to our own mind by the obvious and acknowledged objections which lie against the use of clasps in many, (may we not say in all?) cases of their application. A concise exposition of some of these objections may place in a clearer light the importance of the query above propounded.

In the first place the choice of teeth to which clasps may be attached is limited. No judicious operator will clasp around the incisores or cuspidati for the retention of a single tooth, much less a piece of several teeth. The clasp in this situation will be more or less visible—a serious objection with the patient, and one which sometimes holds good when a first bicuspis is chosen as the tooth to be clasped. The shape of these teeth opposes the retention of clasps, unless placed so far up upon the tooth, as almost inevitably to cause irritation and periosteal inflammation. Lastly, these teeth are more liable than the molares, to have their position deranged either in a lateral or vertical direction, by the traction necessarily exercised by the artificial piece through the medium of its clasps. It is a well known physiological fact one upon the existence of which depends our ability to correct irregular dentures—that a very moderate force, if continuously applied, is sufficient to change the position of a tooth; on the other hand, there unquestionably is such a force exerted, moderate though it may be, in the retention of every piece, tending either to elongate the teeth, especially where they have no antagonist, or to change their position laterally, and in just so far deranging the adaptation of the plate.

Again, with respect to the choice of teeth, there is this objection to the use of the third molares, that unless the plate can be allowed to extend to some distance behind them, which is rarely ever admissible, the weight of the piece, being altogether in front, acts with an undue leverage upon the teeth and brings a strain upon them which must ultimately result in their loss. In the case of the other molares or of the bicuspides, where the plate can extend behind as well as before the line of the teeth clasped, the traction is more immediately vertical, and, where there are teeth below to antagonise, is less likely to prove hurtful.

If, in addition to the above remarks, we take the fact of the greater liability of the first molar to loss from caries or other causes—greater in the proportion of two to one than in the case of the second molaris, or the second bicuspis, and in a still higher proportion compared with the other teeth—we shall find that the number of teeth is limited, to which clasps may be attached with comparative safety. We shall often meet with cases where, in the absence of suitable teeth, we must attach to those liable to the above named objections, or else adopt the other alternative (to which we should gain a very reluctant consent from our patient) and extract them with a view to supply a complete upper set. The difficulty has in a few instances been overcome by the exact adaptation of a plate over the whole palatine arch, being careful that the edge of the plate shall not come too closely against the necks of the teeth still remaining in the mouth. Will not the same purpose be better answered and with less chance of failure by the use of a smaller plate with Cleveland's air chamber?

We have alluded to the physiological principle upon which is based the success of operations, for the regulation of teeth. This success is limited to a certain age, varying in different individuals, from eighteen to twenty-five. After this period, the same force which before could with impunity be applied, will excite periosteal inflammation, and cause the loss of the tooth. The same result often attends the separation of teeth by means of cotton, wood or india rubber, preparatory to filling cavities on their approximal surfaces; and precisely such a result must sometimes happen in teeth to which clasps are attached, whether incisores, cuspidati, bicuspides or molares. This forms our second objection, most applicable to the incisores and cuspidati, but also in some degree to the bicuspides and molares.

A third difficulty is the retention of vitiated mucous or other fluids in the mouth between the clasp and the tooth. This will take place, however closely the clasp may be adapted, and will act more or less injuriously upon the tooth, however careful the patient may be to cleanse the piece daily. Especially is this liable to occur where, from the presence of decay, or other causes, the file has been used. It is the practice of many to file a badly shaped tooth so as better to adapt the clasp, also with the same instrument to separate two teeth to allow the clasp to pass between them. It is unnecessary to observe that such practice materially affects the permanence of the piece by increasing the risk of the loss of the tooth thus treated. We would not be understood as censuring those who adopt this course—it is frequently impossible to do otherwise; but wherever in our own practice such a necessity for the use of the file should occur, we would anxiously inquire if the piece might not be retained in the mouth in some other way than by clasps.

The bicuspides and molares of many persons have crowns so short, or else of so conical a form—larger at the neck than at the base, or *vice versa*—that the adjustment and retention of clasps is exceedingly difficult, and sometimes, unless the file be resorted to, impossible.

Again, the close adaptation of the plate and clasp around the neck of the tooth, is not unfrequently a cause of irritation and subsequent inflammation of the dental and alveolar periosteum. This, like a previous objection, holds with more force against the single than against the double fanged teeth. Yet in cases of peculiar susceptibility, such as are by no means uncommon, it will apply to all. It is to avoid this danger, that the anterior margin of a plate must not be carried too close to such front teeth as may be still remaining. Some dentists seek to obviate the well known difficulty, by soldering a piece of half-round wire to the plate, thus giving it a thicker edge, at the same time bringing it to the very margin of the gum—a very useful and effective measure in case of a *lower* piece, to guard the softer parts against irritation by the otherwise too sharp border; but not so suitable in the present case, because it is not the gum which is irritated so much as the neck of the tooth itself, by the constant pressure, gentle though it may be, of an unyielding metallic plate.

The above remarks are made, not in unreserved condemnation of the use of clasps, but rather as suggestive of the imperfection of the method. However excusable or necessary in any given case, it is, under the most favorable circumstances, an *imperfect* method —imperfect, because it endangers sound natural teeth, which are ever preferable to the best artificial substitutes—imperfect, because it violates fixed physiological principles, and if harmless, is so only by a species of courteous forbearance on the part of nature.

Our art has made rapid improvement, is every day improving, must still greatly improve ere it reaches perfection. What we consider now as defective, is still an improvement upon the past; nor should we condemn and discard any plan till we have found a more unobjectionable substitute. To inquire after such a substitute is the design of the present article. Admitting that there are here and there cases where clasps may be most conveniently applied, and worn for years without injury, we ask—may not the air-chamber or suction plate be substituted, in the great majority of cases, with decided benefit?

Of its size, shape and position, in particular cases, we are not at present prepared to speak—judgment and experience must guide the operator. We think the Journal might be made the medium of much valuable information on this point.

Among the objections to its use, we would briefly notice: First, the interference with distinct articulation, occasioned by the prominence of the plate in the palatine arch. With many this is an important consideration, and if the impediment be considerable and permanent, will prevent the adoption of the plan. Again, the portion of membrane drawn into the opening of the cavity becomes sometimes exceedingly tender and sensitive. This, if persistent, will also prevent the substitution of such a piece for one with clasps. Lastly, the additional expense: it requires more gold, and takes more of that which to the professional man is of more value than gold—his time. These objections we cannot do more at present than simply name. Upon the last of the three we could wish to dwell more at length, not only in its bearing upon this one point, but also as connected with professional services generally: we hope on some future occasion to present to your consideration some remarks upon the "character of dental operations, and their due compensation."

If the above remarks should elicit from any of your numerous readers the detailed result of their individual experience, touching the question proposed, they will fully have answered, Messrs. Editors, the purpose for which they were written. P. H. AUSTEN.

